# IRBAS: An online database to collate, analyze, and synthesize data on the biodiversity and ecology of intermittent rivers worldwide

**DOI:** 10.1002/ece3.2679

**Published:** 2017-01-03

**Authors:** Catherine Leigh, Baptiste Laporte, Núria Bonada, Ken Fritz, Hervé Pella, Eric Sauquet, Klement Tockner, Thibault Datry

**Affiliations:** ^1^ IrsteaUR MALY, centre de Lyon‐VilleurbanneVilleurbanne CedexFrance; ^2^ CESAB‐ FRBImmeuble Henri PoincaréAix‐en‐Provence Cedex 3France; ^3^ Australian Rivers Institute and Griffith School of EnvironmentGriffith UniversityNathanQldAustralia; ^4^ Grup de Recerca Freshwater Ecology and Management (FEM)Departament d'EcologiaInstitut de Recerca de la Biodiversity (IRBio)Universitat de Barcelona (UB)BarcelonaCatalonia/Spain; ^5^ US EPAOffice of Research and DevelopmentNational Exposure Research LaboratoryCincinnatiOHUSA; ^6^ IrsteaUR HHLY, centre de Lyon‐VilleurbanneVilleurbanne CedexFrance; ^7^ IGBLeibniz‐Institute of Freshwater Ecology and Inland Fisheries, and FUInstitute of BiologyFreie Universität BerlinBerlinGermany

**Keywords:** aquatic and terrestrial biodiversity, data sharing, global change, meta‐analysis, temporary river, water management

## Abstract

Key questions dominating contemporary ecological research and management concern interactions between biodiversity, ecosystem processes, and ecosystem services provision in the face of global change. This is particularly salient for freshwater biodiversity and in the context of river drying and flow‐regime change. Rivers that stop flowing and dry, herein intermittent rivers, are globally prevalent and dynamic ecosystems on which the body of research is expanding rapidly, consistent with the era of big data. However, the data encapsulated by this work remain largely fragmented, limiting our ability to answer the key questions beyond a case‐by‐case basis. To this end, the Intermittent River Biodiversity Analysis and Synthesis (IRBAS; http://irbas.cesab.org) project has collated, analyzed, and synthesized data from across the world on the biodiversity and environmental characteristics of intermittent rivers. The IRBAS database integrates and provides free access to these data, contributing to the growing, and global, knowledge base on these ubiquitous and important river systems, for both theoretical and applied advancement. The IRBAS database currently houses over 2000 data samples collected from six countries across three continents, primarily describing aquatic invertebrate taxa inhabiting intermittent rivers during flowing hydrological phases. As such, there is room to expand the biogeographic and taxonomic coverage, for example, through addition of data collected during nonflowing and dry hydrological phases. We encourage contributions and provide guidance on how to contribute and access data. Ultimately, the IRBAS database serves as a portal, storage, standardization, and discovery tool, enabling collation, synthesis, and analysis of data to elucidate patterns in river biodiversity and guide management. Contribution creates high visibility for datasets, facilitating collaboration. The IRBAS database will grow in content as the study of intermittent rivers continues and data retrieval will allow for networking, meta‐analyses, and testing of generalizations across multiple systems, regions, and taxa.

## 1 Introduction

In the era of big data, there is growing interest and need for data collation, synthesis, and access to facilitate scientific networking, discovery, and innovation. This is particularly relevant in the ecological realm given the increasing prevalence and need to address major environmental problems (Hampton et al., 2013). Some of the key questions dominating contemporary discussions and literature on ecological research and management concern the interactions between biodiversity, ecosystem processes, and ecosystem services provision in the face of global change (Cardinale et al., 2012). With freshwater biodiversity in crisis, this is particularly salient for river systems and understanding those interactions in the context of river flow, its modification via human activities (Dudgeon et al., 2006; Vörösmarty et al., 2010), and, increasingly, flow cessation and river drying (Acuña et al., 2014; Datry, Fritz, & Leigh, 2016; Gilvear, Greenwood, Thoms, & Wood, 2016). Which rivers cease flow and dry, either naturally or due to human activities, and how are they distributed across the globe, now and in the future? How does flow cessation and drying influence spatial and temporal patterns in aquatic and terrestrial biodiversity, and how do we monitor, conserve, or restore such biodiversity? Although there is a solid and rapidly growing body of ecological research on rivers that periodically cease flow, herein intermittent rivers for simplicity but described by several terms (e.g., temporary rivers, nonperennial rivers, and ephemeral streams; Datry, Arscott, & Sabater, 2011; Arthington, Bernardo, & Ilhéu, 2014; Leigh, Boulton, et al., 2016), considered among the most common type of running water system in the world, the data encapsulated by this work remain largely fragmented and hence “dark.” As such, our ability to answer the key questions beyond a case‐by‐case basis remains limited.

The Intermittent River Biodiversity Analysis and Synthesis (IRBAS, Datry, Larned, & Tockner, 2014) database is the first of its kind specifically dedicated to the digital storage and provision of biodiversity, and associated hydrological and environmental, data on intermittent rivers worldwide. The IRBAS database (http://irbas.cesab.org) draws on primary datasets, collates data into standardized formats, and is accessible via an online platform where users can contribute, query, and request data. The IRBAS database thus has the capacity to grow in content as the study of intermittent rivers continues and allows data retrieval for meta‐analyses to address questions and test hypotheses across multiple systems, regions, and taxa (Table 1). It also facilitates networking within and among scientists, managers, policymakers, and the public interested or involved in the study and management of river biodiversity (e.g., as represented by networks and aligned projects such as BioFresh, http://project.freshwaterbiodiversity.eu/; Life TRivers, www.lifetrivers.eu; 1000 Intermittent Rivers Project, http://1000_intermittent_rivers_project.irstea.fr; H2020 European COST Action Science and Management of Intermittent Rivers and Ephemeral Streams, SMIRES, http://www.cost.eu/COST_Actions/ca/CA15113).

**Table 1:** Types of metadata stored in the IRBAS database. Asterisks indicate metadata that are available through the IRBAS interface via a map‐based search (Figure 2)

Type	Provision	Details
General metadata	Mandatory	Dataset name*
		Name(s) of the data owner(s) and contributor(s)*
		Contact name(s) and e‐mail(s) for the data provider(s)*
		Data availability status*
		Date the file was created (i.e., when the template was filled and entered into the database)*
		Name(s) of the coder(s) (person(s) who filled the templates with data)
	Optional	Name(s) of the data collector(s)
		Name(s) of the taxonomic identifier(s)
		Name(s) of the research project(s) associated with the dataset
		Any existing citation details (e.g., for the publication(s) associated with the dataset or for the actual dataset itself)*
Site metadata	Mandatory	Name(s) of sampling location(s)
		Latitude(s) and longitude(s) of sampling location(s)*
		Name(s) of river(s) sampled*
		Flow regime (intermittent or perennial) of the sampled river(s)*
		Water regime (permanent or nonpermanent) of the sampled river(s)*
		Name(s) of the catchment(s) and country(ies) in which the sampled river(s) is(are) located*
		Climate (Köppen‐Geiger) classification zone(s) in which the sampled river(s) is(are) located*
		Main land use/land cover category(ies) and level(s) of human modification of the land surrounding the sampling location(s)*
	Optional	Name(s) of discharge, rain, and/or temperature gauging station(s) near or at the sampling location(s)
		Latitude(s) and longitude(s) of discharge, rain, and/or temperature gauging station(s) near or at the sampling location(s)
		Summary information on the long‐term hydrology at the sampling location(s) (e.g., mean annual duration of zero flow)
Biota metadata	Mandatory	Date(s) of sampling*
		Flow state(s) at time of sampling (flowing, not flowing, and dry)*
		Water state(s) at time of sampling (wet and dry)*
		Sampling strategy(ies) and method(s)
		Zone(s) of sampling (e.g., benthic zone)*
		Sampled habitat(s) (e.g., riffle and pool)*
		Number of samples collected at each location*
		Type(s) of biota collected (e.g., aquatic invertebrates)*
		Type(s) of abundance data collected (e.g., counts, presence/absence)*
	Optional	Additional information on sampling methods
Environment metadata (if an Environment template is completed)	Mandatory	Date(s) of sampling*
		Flow state(s) at time of sampling (flowing, not flowing, and dry)*
		Water state(s) at time of sampling (wet and dry)*
		Sampling strategy(ies) and method(s)
		Zone(s) of sampling (e.g., benthic zone)*
		Sampled habitat(s) (e.g., riffle and pool)*
		Number of samples collected at each location*
		Type(s) of measures collected (e.g., waterbody dimensions and water chemistry)*
	Optional	Additional information on sampling methods

In this paper, we describe the IRBAS database, including how users can contribute to it and access data. We outline the key benefits of using and contributing to the database and consider its future applications for fundamental and applied research.

## 2 Database Structure, Modules, and Language

**Figure 1:**
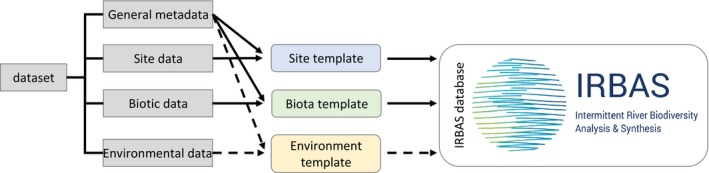
Schematic of the processing and flow of data in the IRBAS database. For each dataset submitted, completion of a Site and Biota template is mandatory (solid arrows) and completion of an Environment template is optional (broken arrows). A dataset must comprise general metadata (e.g., dataset name, data contributor; Table 1) and data collected on sites, biota, and optionally on habitat (environmental data). The Site template is used to insert the site data, including spatial (geo‐locational) data and other metadata about the sampling locations, for example, river names and flow regimes (Table 1). The Biota template is used to insert the biotic sample data (taxonomical inventories of biota) along with metadata on the associated sampling methods and flow status of sites at the time of sampling (Table 1). The Environment template is used to insert the environment sample data (physical and chemical measures) along with metadata on the associated sampling methods and flow status of sites at the time of sampling (Table 1). The general metadata are entered uniformly across each Site, Biota, and Environment templates that encapsulate a unique dataset. IRBAS logo © IRBAS

The IRBAS database is a relational database that houses three main types of data: mandatory (1) site and sampling location information, (2) biotic sample data and methods information, and optional (3) environmental sample data and methods information. Together with general metadata (e.g., data contributor and associated publication information; Table 1), these types of data are each uniquely identified and linked to an individual and uniquely identified dataset, which is entered into the database by a contributor through the use of template files (Figure 1).

The IRBAS database is designed so that it can flexibly handle and represent datasets from different sources, and which may vary, for example, in levels of taxonomic resolution and sampling methods. It is based on the structure of a similarly flexible database, BETSI (http://betsi.cesab.org) for soil invertebrates, which in turn possesses key aspects of other, flexible relational database schema (e.g., as used in genomics research, such as CHADO; http://gmod.org/wiki/Chado) specifically designed to handle complex representations of biological knowledge via the use of controlled vocabulary and modules that logically separate database components. These aspects reduce database complexity while facilitating modification and/or extension in the future.

Several modules handle general metadata on the contributed datasets (Table 1). A dedicated module links source information (e.g., publication details) to each contributed dataset. A second module stores information such as dataset contributor and availability status (i.e., whether the contributor has allowed users full or restricted access to the dataset). A third module stores all other types of general metadata (e.g., the date of entry into the database).

A module is dedicated to storing what we refer to as sample data, allowing us to separate data into biotic (i.e., taxonomic observations of biota) or environmental (e.g., water temperature measures) data. Taxonomic observations are also linked to a taxonomy module which stores information on taxonomic classification. This identifies the rank of each taxon observed (e.g., as family, genus, or species) and allows datasets to include taxa identified at different levels of taxonomic resolution (see *Taxonomy Module* for more information). Each sample within a dataset, whether it is a biotic or an environmental sample, must include information on sampling methods (metadata; Table 1) as well as the actual values of the measures taken (sample data) and must also be linked to a spatial component (i.e., a particular location identified by latitude and longitude in the WGS84 datum) which is managed in a dedicated module and allows samples to be visually located on a map of the world (see *Contributing and Accessing Data*). This spatial information is also considered metadata (Table 1).

The IRBAS database is written in Structured Query Language (SQL) and runs under PostgreSQL, which will allow the option of adding geographical information system (GIS) functionality to the database in the future if desired (via the PostGIS module). Queries are performed as functions written in pl/SQL, via scripting executed through an interface under the content management system (CMS) DRUPAL, written in PHP. This allows data to be checked (e.g., for compatibility with the controlled vocabulary) when inserted into the database and to control what contributors and users of the database can do (e.g., to restrict direct access to the database but allow online querying via an interface). This centralized system, with its consistently organized and described data, can easily be extended into standard interoperable format, such as EML, to enhance discovery and hence reusability of the data.

## 3 Contributing and Accessing Data

**Figure 2:**
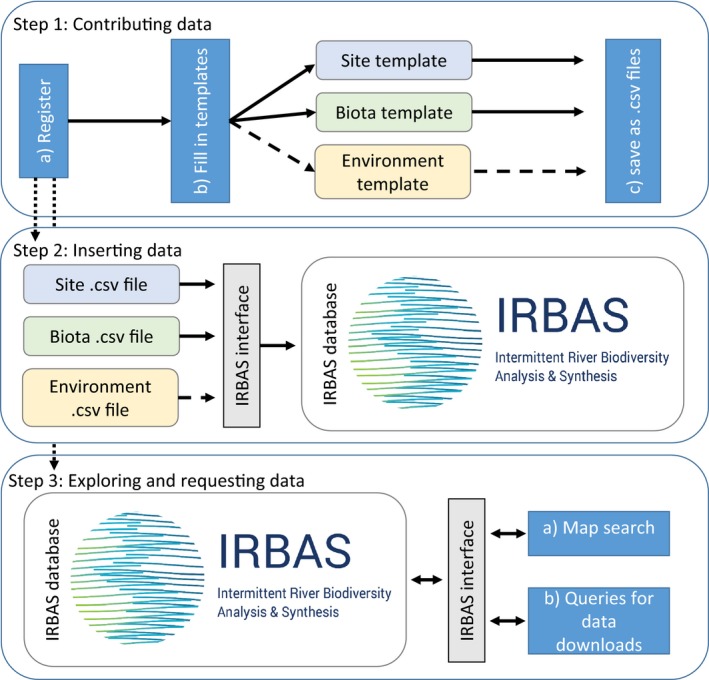
Steps involved in contributing to and accessing data from the IRBAS database. Steps 1 and 2 result in the contribution of a dataset to the IRBAS database (see Figure 1), which requires users to register, fill in templates with data (solid arrows indicate mandatory templates and broken arrows optional templates), save these as.csv files, and then insert them into the IRBAS database via the IRBAS interface. Step 2 involves a quality control and assurance parsing process to ensure data are inserted correctly, completely, and in accordance with the standardization requirements of the database, and the checking of taxonomic nomenclature in the Biota file against the IRBAS taxonomic library (Figure 4). Registered users can explore the database via the interface by zooming in on locations with data presented on a map of the world (Step 3a) and make requests to download data (Step 3b) using a simple check‐box selection procedure that sends specific queries via the interface to the database. IRBAS logo © IRBAS

The IRBAS database is accessible via its online Web interface, hosted by CESAB (Centre de Synthèse et d'Analyse sur la Biodiversité; http://irbas.cesab.org), and includes several pages dedicated to data contribution and access. These pages explain how to:

1 Step 1. contribute data
  - get started as a registered user
  - fill in data entry templates
  - save the completed templates
2 Step 2. insert completed templates into the database
3 Step 3. explore the database and request data
  - via a map interface (identifying locations of samples and extracting metadata)
  - via queries (performing more detailed data requests and extraction) (Figure 2).

### 3.1 Step 1: Contributing data

Contributing data first requires setting up a username and password to become a registered user of the IRBAS database (Step 1a; Figure 2). This allows users to log‐in and gain full access to the database as both a contributor and requester. Registered users wishing to contribute data can download empty template spreadsheets (in the form of tab‐delimited .txt files) that are used to standardize the format, units, and terminology of datasets, samples, and measures (Step 1b; Figure 2).

For each dataset submitted, a completed Site and Biota template must be provided (i.e., completion of these two templates is mandatory); completion of the Environment template is optional (Figure 1). The database is primarily a biodiversity database, but environmental data are welcome and increase the value and usefulness of the data contributed and the versatility of the database.

Mandatory:

1. Site: used to insert spatial (geo‐locational) data and other metadata on the sampling sites and study area, such as river names and flow regimes (i.e., intermittent or perennial)
2. Biota: used to insert biotic sample data (taxonomical inventories of biota, including count, presence/absence, biomass, or other biological measures) along with metadata on the associated sampling methods and flow status of sites at the time of sampling (i.e., flowing, not flowing, or dry)

Optional:

Environment: used to insert physical and chemical sample data along with metadata on the associated sampling methods and flow status of sites at the time of sampling (i.e., flowing, not flowing, or dry).

These templates, along with a set of prefilled examples, and a help guide for their completion, are downloadable via the interface. Each template also contains fields for general metadata which detail the associated dataset name, data contributor, associated publication citation details, data availability status, etc. (Table 1). The general metadata must be uniform across each Site, Biota, and Environment templates that encapsulate a unique dataset (Figure 1). The data availability status is decided by the contributor and indicates whether all of the data are available or whether access is restricted to metadata only (see also *Important Considerations*). Contributors must save their completed templates (Step 1c; Figure 2) before insertion into the database.

### 3.2 Step 2: Inserting data

Saved, completed templates are inserted into the database via the interface's file‐selection dialog box (Step 2; Figure 2). Step 2 involves a quality control and assurance parsing process to ensure data are entered correctly, completely, and in accordance with the standardization requirements [e.g., mandatory fields are filled, taxonomy is acceptable (see *Taxonomy Module*), data values are not impossible, and sample identifiers are unique]. Where errors are detected, the contributor is given information, via the interface, which will allow them to detect and correct the errors, and resave and reinsert their files. This process continues until no further errors are detected at which point the contributor is informed that the files have been inserted successfully.

### 3.3 Step 3: Exploring the database and requesting data

**Figure 3:**
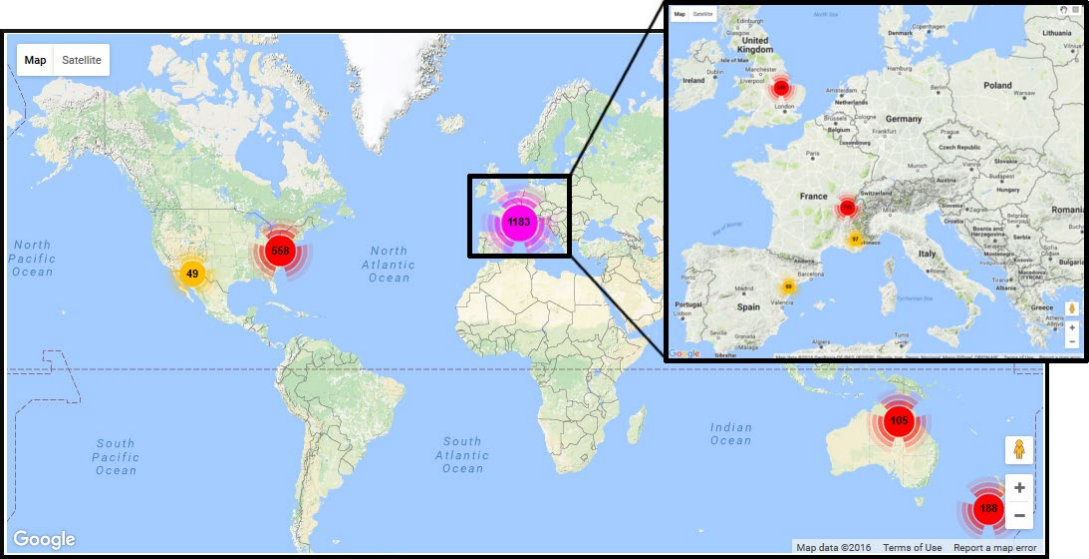
Geographical coverage and numbers of samples with biodiversity or environmental data collected from intermittent and nearby perennial rivers, stored in the IRBAS database as of August 2016. The IRBAS database allows users to contribute and access data and will thus continue to grow in content and value as a research and networking tool. Map data: ©2016 Google

All registered users can explore the database and access data via a map (Step 3a; Figures 2 and 3) or via queries (Step 3b). The map shows the locations in the world of all samples across all datasets in the database. Users can zoom in and out to identify specific locations where samples are present and to access summary metadata on these samples and locations (Table 1). Queries follow a simple check‐box procedure and allow users to make generic or specific data requests via the interface. For example, users may be interested in data on fish only, or from certain time spans, rivers or regions, or in certain datasets only. Results are extracted from the database and provided to users as downloadable (tab‐delimited) .csv files.

## 4 Taxonomy Module

**Figure 4:**
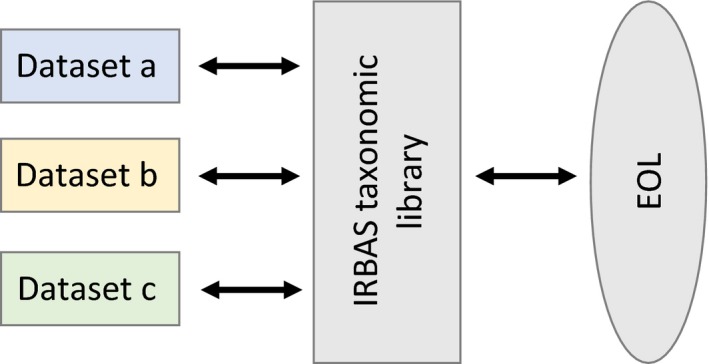
The IRBAS database uses Encyclopedia of Life (EOL; http://www.eol.org/) as its authoritative resource for taxonomic nomenclature and classification hierarchies. All names of taxa supplied by contributors are parsed via a list of names stored on the database (the IRBAS taxonomic library), recognized by EOL, before datasets are inserted into the IRBAS database

To ensure taxa stored in the IRBAS database are named according to the latest international conventions, the IRBAS database uses Encyclopedia of Life (hereafter EOL; http://www.eol.org/) as its authoritative resource for taxonomic nomenclature and classification hierarchies. The taxonomic and geographical coverage of EOL is not limited to particular countries, continents, or hemispheres, or to particular groups of organisms (e.g., invertebrates, fish, or plants), thus matching the global scope of the IRBAS database. All names of taxa supplied by contributors are parsed via a list of names stored on the database (the IRBAS taxonomic library), recognized by EOL, before datasets are inserted (Figure 4).

We compiled the library by first identifying families of taxa from around the world commonly found in rivers, wetlands, and riparian zones. All taxa within these families and their associated classification ranks within the taxonomic hierarchies listed in EOL were then entered into the database using a PHP script and the EOL application programming interface (API) (http://eol.org/info/api_overview), which essentially embeds the functionality of EOL into the taxonomy module of the IRBAS database. The resulting, relational list of taxa (>600,000) and their ranks is likely to cover the majority of taxa encountered in contributed datasets. During dataset insertion, the taxonomic nomenclature used in the completed Biota files of contributors is checked against this EOL‐generated library, and if errors are encountered, the contributor is alerted following the procedure outlined in Step 2 above. Taxa can also be stored locally, being added to the IRBAS taxonomic library by the administrator when and if required (with liaison with EOL if required).

## 5 Database Content

As of August 2016, the IRBAS database houses data from 11 contributed datasets, associated with over 2,000 individual samples of biological, hydrological, and environmental data and over 400 taxa. These data were collected from intermittent rivers (and in some cases also from nearby perennial rivers) in North America (USA, 3 datasets), Europe (France, Spain, and UK; 3, 1 and 1 datasets, respectively), and Oceania (Australia and New Zealand; 1 and 2 datasets, respectively; Figure 3). The database currently lacks data from the continents of Africa, Asia, and South America and from rivers in tropical (D) and polar‐alpine (E) climate zones (according to the Köppen‐Geiger climate classification). The majority of sampling sites are in temperate climate zones (60%) and on intermittent rivers (67%). The database also includes data collected from perennial rivers and perennial reaches of intermittent rivers. This is because many ecological studies of intermittent rivers seek to make comparisons with perennial rivers (asking questions such as “Are biological communities of intermittent river reaches subsets of those of nearby perennial river reaches?”) to better understand how flow intermittence acts as a hydrological determinant of river biodiversity (e.g., Datry, Larned, Fritz, et al., 2014; Leigh & Datry, 2016). Hence, the IRBAS database accepts datasets comprising data collected from both intermittent and perennial rivers.

Biotic samples currently housed in the database primarily describe aquatic invertebrate communities (80% of biotic samples). Fish and terrestrial invertebrate communities are represented to a lesser extent (7% and 8% of biotic samples, respectively), and to date, there are no plant community samples represented. Biota have been sampled primarily from rivers during wet hydrological phases (85% of biotic samples collected from flowing reaches and 14% from nonflowing, isolated pools of surface water), with only five biotic samples collected from dry riverbeds. This reflects the historical focus on understanding biodiversity patterns in perennially flowing rivers, with intermittent rivers sampled most commonly when they were flowing (or at least containing surface water in isolated pools; Leigh, Boulton, et al., 2016). Growing interest in the dry (terrestrial) phases of intermittent rivers and understanding these systems as coupled aquatic‐terrestrial ecosystems will help to rectify these gaps (Corti & Datry, 2016; Datry, Pella, Leigh, Bonada, & Hugueny, 2016; Steward, von Schiller, Tockner, Marshall, & Bunn, 2012).

## 6 Benefits of Contributing and Accessing Data

The IRBAS database was compiled with the view, espoused by collaborative research centers such as CESAB (http://www.cesab.org/), National Center for Ecological Analysis and Synthesis (NCEAS; https://www.nceas.ucsb.edu/), and Australian Centre for Ecological Analysis and Synthesis (ACEAS; http://www.aceas.org.au/), that answers to important ecosystem, ecological, and biodiversity questions can be elicited through the assembly of multiple and heterogeneous existing datasets, facilitating collaboration and analyses, and insights beyond those possible from the use of data collected by individual studies or research and management programmes. Recent meta‐analyses and syntheses of biodiversity data from intermittent rivers around the world (Datry, Larned, Fritz, et al., 2014; Leigh & Datry, 2016; Leigh, Stubbington, Sheldon, & Boulton, 2013; Leigh, Bonada, et al., 2016) demonstrate the value of collating, standardizing, and analyzing data from disparate sources; in fact, many of the individual datasets compiled and synthesized for these studies are now available via the IRBAS database. These studies have deepened understanding of the resistance and resilience of aquatic biota to flow intermittence and identified general relationships between hydrological and environmental drivers and community responses, a major goal of ecological and biodiversity research. Further insights will no doubt come from new analyses and continued synthesis of data from across the globe facilitated by the IRBAS database to address the myriad of questions and challenges that intermittent river systems, respectively, inspire and pose (Datry, Fritz, et al. 2016; Datry, Larned, & Tockner, 2014; Table 2).

**Table 2:** Ten research questions the IRBAS database is helping to address or may help to address in the future

Top research questions	
1.	Are there predicable, cyclic trajectories of change in community composition that can be matched to cycles of hydrological phases in intermittent rivers?
2.	To what extent do trajectories of population and community change depend on antecedent, local and regional physical, and chemical and biological conditions, both individually and interactively?
3.	How do food webs assemble and disassemble in intermittent rivers as they cycle through the different hydrological phases?
4.	How are taxonomic, functional, phylogenetic, and genetic diversity related to each other and to ecosystem diversity (e.g., measured as spatiotemporal habitat heterogeneity) in intermittent rivers, and do these relationships vary across spatial and temporal scales?
5.	Are intermittent rivers equivalent to the sum of uncoupled aquatic and terrestrial ecosystems in terms of biodiversity or are they more than that?
6.	How congruent are the responses of terrestrial and aquatic biota of intermittent rivers to hydrological, physical, chemical, and biological changes in their environment?
7.	Are biological responses to changes in the environment and to stresses such as floods in intermittent rivers the same as observed in perennial rivers?
8.	Are the biological metrics and indicators currently used to access ecological condition of perennial rivers suitable for intermittent rivers, and if so, where and when?
9.	Are there predictable differences in community diversity and composition between natural and human‐induced intermittent rivers?
10.	How are intermittent rivers and their biota responding to global change, and what do we predict for the future?

Through its visibility, the database portal will also raise awareness of the global prevalence and ecological significance of intermittent rivers for scientists and water managers. Biological and ecological information on intermittent rivers is being sought increasingly by managers and policymakers faced with increasing flow intermittence, resulting from climate change and growing demands for freshwater, and the monitoring and management challenges this presents (Datry, Fritz, & Leigh, 2016). Observed and projected shifts from perennial to intermittent flow regimes and increased duration and frequency of drying in intermittent rivers have thus heightened the need for a centralized and open‐access database such as the IRBAS database.

By nature of its open access, the IRBAS database will promote synergies among research groups worldwide. It will also build on previous freshwater biodiversity data synthesis efforts at the European scale and beyond, for example, via links to BioFresh (http://data.freshwaterbiodiversity.eu/) and GBIF (http://www.gbif.org/), and will continue to grow both in terms of its user and dataset numbers. For example, the database will be expanded and used by a consortium of 150 researchers involved in the SMIRES Cost Action.

## 7 Important Considerations

The IRBAS consortium has applied the Creative Commons CC‐By 4.0 (https://creativecommons.org/licenses/by/4.0/) approach to protect the data and ensure that data providers are properly acknowledged by users. Access to the database is password protected and to ensure users that the data they contribute are held securely: (1) Data from the IRBAS database are not to be used for any commercial purpose without permission from the data contributor(s) and (2) users accept all risks and responsibility for losses, damages, costs, and other consequences (direct or indirect) resulting directly or indirectly from using the interface and any information or material available from it.

Any use of data from the IRBAS database must attribute credit to the IRBAS consortium (by citing this IRBAS database publication along with the IRBAS project publication Datry, Larned, & Tockner, 2014) and the original data contributor(s). Use of the data must also include citation of the original source(s) [e.g., journal publication(s)] describing the collection and presentation of the relevant datasets. Citation details are available when accessing data via the interface to the IRBAS database (http://irbas.cesab.org).

Users need also to be aware that the data housed within the database are limited to those encapsulated by the fields and standardized formats of the Site, Biota, and Environment templates. There is capacity, however, to expand, adapt, or improve the existing database owing to the underlying, flexible nature of the database structure and its use of modules and a controlled vocabulary. Essentially, new functionalities and data types could be added in the future without the need for major redevelopment. This, for example, could include the addition of new modules linked to the taxonomy module that house molecular systematics and species traits information.

The IRBAS database is currently incomplete in terms of its biogeographic and taxonomic coverage (see *Database Content*); this paper serves as a call to researchers to share their data, from individual and large‐scale investigative studies, and thus play an active role in expanding the database. This will not only help to close gaps in coverage but also facilitate networking and ultimately deliver a deeper understanding and appreciation of the biodiversity of intermittent rivers.

## 8 Concluding Remarks

The IRBAS database acts as both a data portal and discovery tool, facilitating the synthesis and analysis of data to elucidate patterns in and controls on intermittent river biodiversity. Contributing data, and providing them in the standardized formats generated by the database, not only increases data visibility but also useability. As the IRBAS database grows, we expect to see new insights arising from future, previously impossible and unimagined, meta‐analyses and for collaborative research initiatives to continuously evolve. This will allow generalizations to be tested across multiple systems, regions, and taxa and better equip scientists and managers with the data and knowledge they need to study, conserve, or restore river ecosystems and biodiversity. We heartily encourage contributions of data from around the world.

## Conflict of Interest

None declared.
